# T162. LEFT PREFRONTAL GYRIFICATION IN HEALTHY SUBJECTS ASSOCIATED WITH SUBCLINICAL PSYCHOTICISM PHENOTYPE

**DOI:** 10.1093/schbul/sby016.438

**Published:** 2018-04-01

**Authors:** Igor Nenadic, Katharina Frisch, Bianca Besteher, Christian Gaser

**Affiliations:** 1 Philipps-Universität Marburg - UKGM; 2 Jena University Hospital

## Abstract

**Background:**

Recent studies have shown aberrant gyrification, i.e. folding of the cortical surface, as a marker of disturbed neocortical development in schizophrenia. Unlike more commonly used markers like voxel-based morphometry, these parameters might be more sensitive to early neurodevelopmental pathologies. It is unclear, however, whether such structural changes might be evident across the schizophrenia spectrum, involving at-risk subjects as well as even healthy subjects with subclinical or attenuated psychotic(-like) symptoms

**Methods:**

We analysed high-resolution MRI scans (3 Tesla, T1-weighted MPRAGE, 1x1x1mm resolution) from n=177 healthy subjects with no current or previous psychiatric condition recruited from the local community. Subjects completed the SCL90R, a general symptom checklist (i.e. self-rating of symptoms), which includes subscales for psychoticism (with subclinical psychotic/-like symptoms) and paranoid ideation. We used the CAT12 toolbox to analyse both gyrification using the absolute mean curvature approach (Luders et al., NeuroImage 2006, as well as cortical thickness and voxel-based morphometry (VBM).

**Results:**

Correcting for effects of age and gender, we found a significant negative correlation between SCL90R psychoticism scores and gyrification in a left prefrontal / frontopolar cluster, but no similar finding for wither cortical thickness analysis nor analyses of the paranoid ideation subscale.

**Discussion:**

Our results suggest that prefrontal gyrification might be a marker for psychotic phenotypes spanning a spectrum from subclinical symptom expression to frank psychosis. This association seems linked to gyrification (rather than other markers of brain structure), which would suggest a relative specificity. Hence, this would be consistent with the assumption that gyrification is related to early neurodevelopmental effects, which lead to liabity to experiencing psychotic symptoms later in life, and might thus serve as an imaging phenotype for early risk detection and intervention in high-risk groups.

